# Cognitive subgroups and their longitudinal trajectories in bipolar disorder

**DOI:** 10.1111/acps.13460

**Published:** 2022-06-25

**Authors:** Tobin J. Ehrlich, Kelly A. Ryan, Katherine E. Burdick, Scott A. Langenecker, Melvin G. McInnis, David F. Marshall

**Affiliations:** ^1^ Heinz C Prechter Bipolar Research Program, Eisenberg Family Depression Center, and Department of Psychiatry University of Michigan Ann Arbor Michigan USA; ^2^ Department of Psychiatry, Brigham and Women's Hospital Harvard Medical School Boston Massachusetts USA; ^3^ Department of Psychiatry University of Utah Salt Lake City Utah USA

**Keywords:** bipolar disorder, cognition, hierarchical clustering, longitudinal modeling

## Abstract

**Introduction:**

Cognitive functioning in bipolar disorder is heterogeneous with evidence for multiple subgroups. However, cognitive subgroup change patterns over time remains unknown. While prior work suggests minimal differences in cognitive functioning patterns over time between those with bipolar disorder and controls, group‐based analyses may obscure unique subgroup‐based changes.

**Material and Methods:**

Participants diagnosed with bipolar disorder (I, II, NOS; *n* = 568) and unaffected controls (*n* = 234) completed baseline, one‐ and five‐year neuropsychological assessments. Data reduction techniques were used to limit the number of neuropsychological variables. Bipolar disorder participant baseline neuropsychological data were entered into hierarchical cluster analyses and resultant clusters were entered in multilevel models, which tested for differences in baseline and longitudinal cognitive changes in cognition among the cluster groups and with controls.

**Results:**

Results were consistent with bipolar disorder participants forming three subgroups with high (*n* = 209), mid (*n* = 259), and low (*n* = 100) cognition. These groups were associated with unique clinical characteristics. Multilevel models demonstrated that over a five‐year period, the low group improved, relative to the high and mid groups, and with controls, in auditory memory. Over the five‐year period, the mid group, in comparison with the high group, improved in visual memory; additionally, the high group remained stable, in comparison with a slight decline in the control group, in inhibitory control.

**Conclusion:**

These results demonstrate that cognition‐based subgroups of bipolar disorder participants have minimal differences in their longitudinal course in relation to each other and with unaffected controls.

**Significant outcomes**

- The majority of participants with bipolar disorder have average cognitive functioning with a relative minority demonstrating significantly lower global cognitive functioning.
- Cognitive based subgroups are unique in their demographic, clinical, and mood characteristics but do not significantly differ in their rate of change over a five‐year period.

**Limitations**

- The sample may not generalize to the larger population of people with bipolar disorder as participants tended to be middle aged, have higher than average educational attainment, higher than average estimated intelligence, and were capable of staying in a longitudinal study.
- A five‐year follow‐up may not be of sufficient duration to observe declines in cognition.

## 1 INTRODUCTION

Cognitive functioning in bipolar disorder (BP) is heterogeneous, spanning intact cognitive functioning to significant global impairments.^1^, ^2^, ^3^ This heterogeneity in cognition appears to be reflected in distinct groups within the BP population, suggesting that BP can be characterized by cognitive subgroups. Studies of cognitive subgroups in BP generally show an average functioning group, a selectively impaired group, and a globally impaired group.^4^, ^5^, ^6^, ^7^, ^8^ The selectively and globally impaired groups typically present with adverse clinical or demographic characteristics, such as less education, increased depression severity, or use of medications with known cognitive side effects. This heterogeneity in cognitive functioning and the associated adverse clinical characteristics, combined with the importance of cognition in everyday functioning,^9^ highlights the impact of impaired cognitive functioning of people with BP.

A parallel line of research that has similarly provided greater understanding of cognitive functioning in BP is the change over time. Characterizing the longitudinal change patterns in cognition will help to prioritize unique treatment needs of people with BP.^10^, ^11^ Despite the prevailing belief that BP may be neuroprogressive, with changes in brain structure and function accumulating with illness recurrence,^12^ the plethora of data from longitudinal studies do not support this, suggesting that people with BP do not have increased rates of cognitive decline as compared with controls.^13^, ^14^, ^15^ Recent follow‐up studies of five or more years have supported similar rates of cognitive decline in BP as compared with controls.^10^, ^14^, ^16^ However, studies of longitudinal changes in cognition have primarily evaluated BP based upon diagnosis (i.e., I vs. II) or as a uniform group, potentially masking unique longitudinal changes in cognitive subgroups. Cognitive subgroups not only provide a novel means to study the heterogeneity of cognitive functioning in BP cross‐sectionally, but these subtypes may provide unique means to delineate cognitive trajectories and associated treatment needs.

Consistent with prior studies, we (1) hypothesize that our sample of individuals with BP will fit into three distinct groups that will have unique clinical characteristics. Additionally, we (2) hypothesize that there will be minimal differences in the five‐year longitudinal course of cognition between those with higher cognitive functioning as compared with controls, while (3) those with lower cognitive functioning will diverge from those with higher cognitive functioning and controls.

### 1.1 Aims of the study

The goals of the current study are twofold. First, we aim to identify cognitive subgroups in a large, thoroughly characterized, sample of individuals diagnosed with BP and to describe differential clinical characteristics. Second, we will use the derived cognitive subgroups to evaluate their five‐year longitudinal cognitive course. Longitudinally, our interest lies in how the cognitive subgroups may demonstrate divergent longitudinal trajectories from each other and with controls.

## 2 MATERIALS AND METHODS

### 2.1 Participants

Participants were enrolled in the Heinz C. Prechter Longitudinal Study of BP at the University of Michigan, an observational cohort study of BP gathering phenotypic and biological data, approved by the University of Michigan Institution Review Board, for full study details see McInnis et al.^17^ All participants provided signed informed consent and were financially compensated for their participation. Recruitment into the longitudinal study was through advertisements on the internet, in the newspaper, in an outpatient specialty psychiatric clinic, community mental health centers, community outreach events, and in an inpatient psychiatric unit. Exclusion criteria included having a diagnosis of schizophrenia or active and current substance abuse or dependence or a medical illness specifically associated with depression (including but not limited to: terminal cancers, Cushing's disease, or stroke) at the time of enrollment. At study baseline, participants underwent the Diagnostic Interview for Genetic Studies (DIGS) on an outpatient basis. Diagnoses of BP or control (defined as no history of a mental health disorder) were confirmed through a two‐person review (PhD and/or MD) and consensus of the DIGS diagnostic data. The sample for the current analyses included a total of 802 participants, 568 diagnosed with BP and 234 controls at the baseline evaluation. Updated diagnostic data was available at 4 years post study entry. Nine BP participants had a change in their diagnosis, further details are described in the online Appendices (Data S1).

### 2.2 Neuropsychological assessment and cognitive factors

Neuropsychological testing was administered by trained staff at baseline, one‐year, and five‐years after study enrollment as part of the longitudinal study protocol.^17^ Each participant was randomized to one of two neuropsychological test batteries that included alternate forms (as available) at each testing time point, which were then alternated between testing occasions. Methods similar to this have been shown to attenuate practice effects.^18^ Current (prior 2 weeks) euthymic state was not required to complete the neuropsychological assessments, either for baseline or follow‐ups. On average, BP participants were experiencing minimal to mild symptoms of depression and minimal symptoms of mania at the baseline, one‐ and five‐year neuropsychological assessments (see Table 1). This research‐defined test battery was chosen to focus on cognitive domains known to be adversely affected in BP. To reduce the number of neuropsychological data points, factor analytic techniques were used. Briefly, test scores with lower scores reflecting better performance were inverted, such that higher scores indicate better performance for all cognitive metrics, all raw test scores were then z‐transformed (based upon the total control sample mean scores), and then categorized based upon conceptual and theoretical knowledge of factor structures. The cognitive factors used in this study were originally created by Langenecker et al.^19^ with a combination of theoretically derived confirmatory and exploratory factor analyses. Confirmatory factor analyses were applied to auditory memory, visual memory, fine motor dexterity, and emotion processing test subsets based upon theoretical and prior empirical data. Exploratory factor analysis was applied to tests that traditionally are broadly considered under the umbrella term of executive functions. This exploratory factor analysis resulted in four additional factors: verbal fluency and processing speed, conceptual‐reasoning and set‐shifting, processing speed with inference resolution, and inhibitory control. In order to focus on core cognitive processes, the fine motor dexterity and emotion processing factors were not used in the present study. For the purposes of the present study all cognitive data was age corrected. Age correction was completed with available normative data for each of the subtest raw scores. The age corrected subtest scores were then combined into the original six factors. Details of the tests included in each of the six cognitive factors is found in the online Appendices (Data S1).

**TABLE 1:** Demographic and clinical characteristics

	Total BP *n* = 568	(1) High *n* = 209	(2) Mid *n* = 259	(3) Low *n* = 100	(4) Controls *n* = 234	Sig. diff.
Age	38.80 (13.39)	39.72 (14.14)	38.20 (12.98)	38.45 (12.86)	35.03 (15.53)	1 > 4, 2 > 4, 3 > 4
Female	368 (65%)	113 (64%)	169 (65%)	66 (66%)	136 (59%)	n.s.
Education	15.18 (2.24)	15.64 (2.12)	15.17 (2.23)	14.27 (2.26)	15.81 (2.17)	1 > 2, 1 > 3, 2 > 3, 4 > 2, 4 > 3
Estimated intelligence	108.92 (12.50)	115.43 (8.85)	108.49 (10.77)	95.85 (13.16)	112.82 (11.74)	1 > 2, 1 > 3, 1 > 4, 2 > 3, 4 > 2, 4 > 3
1‐year neuropsychological evaluation (months since baseline)	13.24 (3.01)	13.03 (2.57)	13.31 (3.46)	13.54 (2.54)	12.79 (2.40)	n.s.
5‐year neuropsychological evaluation (months since baseline)	61.90 (3.42)	62.38 (3.69)	61.54 (3.20)	61.71 (3.22)	61.25 (3.95)	n.s.
Age of onset	17.16 (7.70)	16.63 (7.59)	17.19 (7.45)	18.20 (8.52)		n.s.
Baseline depression severity	9.52 (6.24)	8.28 (5.40)	9.81 (6.59)	11.33 (6.45)		3 > 1, 3 > 2, 2 > 1
1‐year depression severity	8.50 (5.86)	7.70 (5.29)	8.50 (6.07)	10.44 (6.19)		3 > 1, 3 > 2
5‐year depression severity	8.00 (5.74)	7.32 (5.66)	8.67 (5.80)	7.79 (5.70)		n.s.
Baseline mania severity	3.56 (3.91)	3.24 (3.57)	3.57 (4.05)	4.15 (4.13)		n.s.
1‐year mania severity	3.30 (4.03)	2.83 (3.42)	3.47 (4.35)	3.93 (4.34)		n.s.
5‐year mania severity	3.20 (4.14)	3.18 (4.07)	2.96 (3.44)	4.07 (6.09)		n.s.
Childhood trauma	48.48 (19.77)	47.33 (19.81)	46.49 (18.39)	59.56 (21.74)		3 > 1, 3 > 2
Psychiatric hospitalizations	2.54 (3.56)	1.67 (2.48)	2.73 (3.66)	4.20 (4.93)		3 > 1
Number of comorbid psychiatric diagnoses	2.30 (2.15)	2.11 (2.12)	2.36 (2.24)	2.57 (1.95)		n.s.
Body mass index	32.69 (8.96)	32.69 (8.69)	32.19 (8.86)	34.14 (9.83)		n.s.
Number of medical comorbidities	4.72 (4.51)	4.99 (4.69)	4.55 (4.48)	4.55 (4.25)		n.s.
Number of manic episodes per year	0.43 (0.99)	0.39 (0.72)	0.44 (1.19)	0.52 (0.92)		n.s.
Number of depressive episodes per year	1.16 (1.54)	1.15 (1.52)	1.13 (1.47)	1.26 (1.76)		n.s.
Medication load	2.38 (2.06)	2.32 (1.92)	2.51 (2.09)	2.15 (2.26)		n.s.
History of a suicide attempt	304 (54%)	110 (53%)	144 (57%)	50 (50%)		n.s.
History of a substance use diagnosis	316 (56%)	100 (48%)*	148 (57%)	68 (68%)*		
Bipolar‐I diagnosis	390 (69%)	131 (63%)*	185 (71%)	74 (74%)		
Bipolar‐NOS diagnosis	61 (11%)	24 (12%)	24 (9%)	13 (13%)		n.s.
Antipsychotic use	171 (31%)	54 (27%)	83 (33%)	34 (36%)		n.s.
History of psychosis	246 (48%)	79 (41%)*	117 (49%)	50 (58%)*		

*Note*: Data are provided as mean (standard deviation) or *n* (percentage), as appropriate. The significant differences column provides the results of ANOVA group comparisons for the (1) BP high performance group, (2) BP mid performance group, (3) BP low performance group, and (4) controls with significance set at *p* < 0.05. The Total BP column is provided for descriptive purposes only and are not included in the analyses.

* Significant χ ^2^ results (*p* < 0.05).

At study baseline, the Wechsler Abbreviated Scale of Intelligence, Second Edition^20^ was administered as an estimate of intellectual functioning.

### 2.3 Clinical variables

Clinical variables were collected during entry into the longitudinal study.^17^ These variables included age, education, sex, number of psychiatric hospitalizations, history of psychosis, history of a substance use disorder, number of comorbid psychiatric disorders, BP subtype (I, II, NOS), age of BP onset, history of a suicide attempt, body mass index (BMI), number of medical comorbidities, antipsychotic medication use, and medication load (additive value of different classes of prescribed medications).^21^, ^22^ The clinician rated Hamilton Depression Rating Scale – 17‐item (HAMD) was used to evaluate depression severity^23^ and the Young Mania Rating Scale (YMRS) was used to evaluate mania severity^24^ at each time point. The total score of the Childhood Trauma Questionnaire (CTQ) was used to evaluate childhood trauma severity.^25^

### 2.4 Statistical analyses

IBM SPSS 27 was used for all statistical analyses. Baseline data only for the six cognitive factor scores for each participant were entered in a hierarchical cluster analysis. The agglomerative approach used was the squared Euclidean distance and Ward linkage criteria. The dendrogram was visually inspected to identify the appropriate number of clusters and each participant was then assigned to their identified cluster. To evaluate for cluster stability, this process was repeated with a random split‐half sample. The total sample results were further confirmed through a discriminant function analysis (DFA). The DFA identifies which factor scores best discriminate between the clusters and how well these factor scores predict cluster membership. Additionally, a leave‐one‐out cross validation technique was used to confirm the stability of the clusters. To understand the characteristics of the clusters, differences in cognitive and clinical variables were evaluated with planned analysis of variance (ANOVA) or chi‐squared (χ ^2^), as appropriate. Differences in baseline cognitive performance were adjusted for multiple comparisons with a Bonferroni correction (six cognitive factors at an error rate of 0.05, yielding *p* < 0.008). The resultant groups were evaluated for proportional differences in participant attrition with a χ ^2^ analysis between the baseline and five‐year neuropsychological evaluations. Differences in depression and mania severity at the time of the neuropsychological evaluations among the derived groups and between the timepoints were evaluated with ANOVA analyses. Differences among the groups in the number of months between the baseline, one‐ and five‐year neuropsychological evaluations was also evaluated with an ANOVA analysis.

Multilevel modeling was utilized to evaluate the baseline, one‐ and five‐year longitudinal factor scores for these cluster groups. Multilevel modeling minimizes the potentially biasing effects of unbalanced data and accounts for autocorrelations inherent to longitudinal data analyses.^26^ The multilevel models were run with the MIXED procedure with maximum likelihood estimation. Singer and Willet^26^ suggest linear modeling when three waves of data are available. Additionally, the authors suggest examining empirical growth plots to evaluate if these plots support linearity. Therefore, consistent with the author suggestion and evidence from the empirical growth plots, linear modeling was undertaken. Time was centered to zero to adjust the baseline factor scores to the intercept. Unconditional growth models were first run to identify if there were significant differences from zero in baseline or rate of change for the total sample for each of the six cognitive factors. Each of the six cognitive factors were then individually evaluated for differences in baseline and rate of change among the hierarchical cluster groupings and with controls. The between group comparisons were modeled with binary contrasts comparing each pair of clusters and with controls.

## 3 RESULTS

### 3.1 Participant characteristics

Participants include 568 individuals diagnosed with BP, 390 with BP‐I, 117 with BP‐II, and 61 with BP‐NOS. On average, those diagnosed with BP were 39 years of age, had 15 years of education, an average estimated IQ of 109, and were 65% female. The controls consist of 234 participants who, on average, were 35 years of age, had 16 years of education, an average estimated IQ of 112, and were 59% female. Demographic characteristics for BP and controls are found in Table 1.

### 3.2 Cognitive cluster results

**FIGURE 1:**
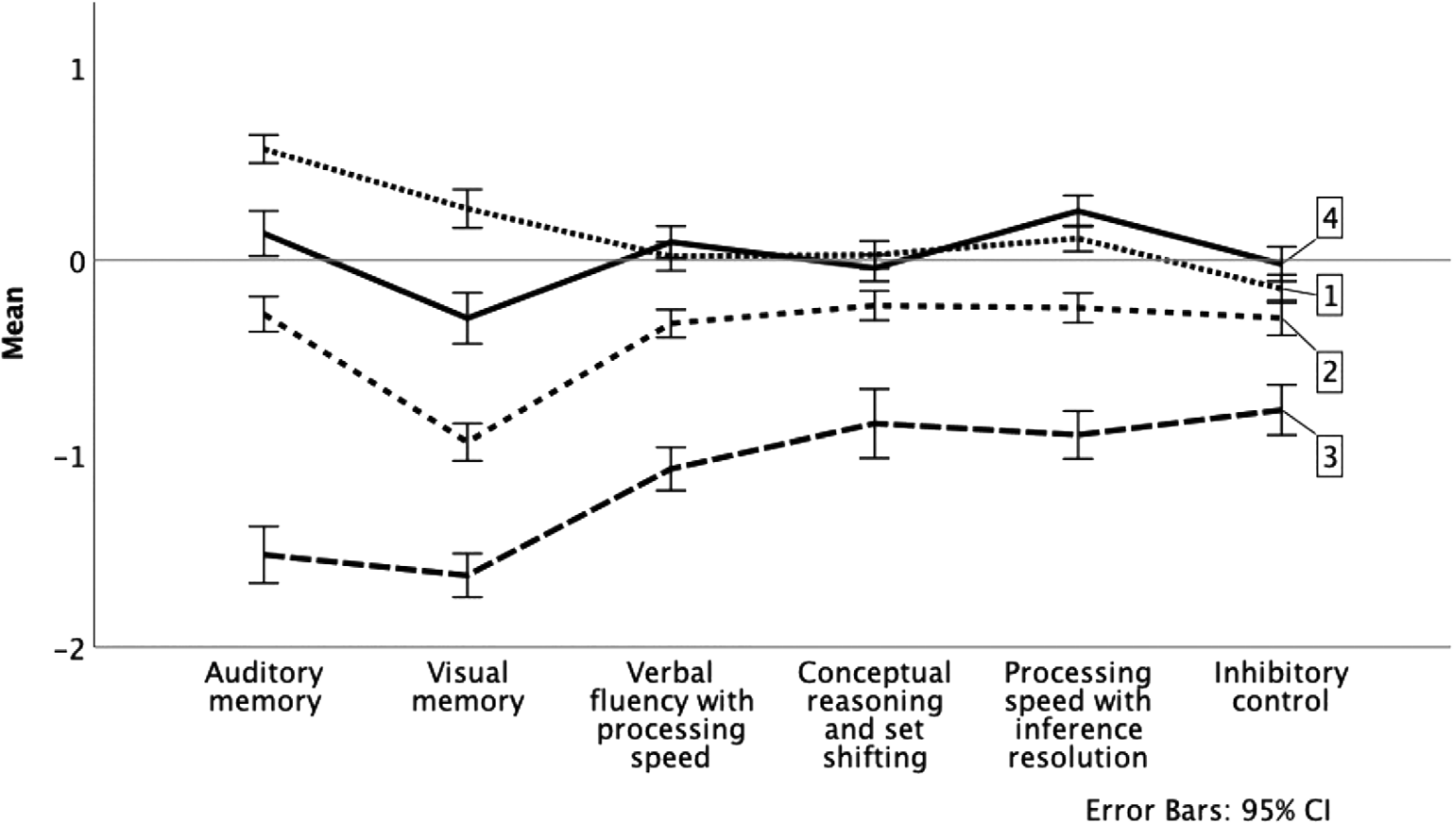
Baseline cognitive factor means for the three clusters and controls. 1 = high performance group, 2 = mid performance group, 3 = low performance group, 4 = control group

Inspection of the dendrogram suggested a three‐cluster solution with 209 (36.8%) in a high performance group, 259 (45.6%) in a mid‐performance group, and 100 (17.6%) in a low performance group. The results of the split half sample hierarchical cluster analysis (*n* = 306) indicated good stability of the groups with a 5.2%, 8.9%, and 10.9% change in cluster size for the high, mid, and low performance groups, respectively. The DFA results for the six factor scores generated two functions that separated the three clusters. The first function explained 98.0% of the variance (Wilks' λ = 0.198, *p* < 0.001) and the second function explained the remaining 2.0% of the variance (Wilks' λ = 0.931, *p* < 0.001). The largest correlational coefficients were for the auditory memory factor (*r* = 0.76) and the visual memory factor (*r* = 0.74), suggesting that these two factors contribute the most to the differentiation of the clusters. Based upon the DFA, the overall correct classification rate was 88.2%. The correct classification rates per group were 91.9% for the high performance group, 82.6% for the mid performance group, and 95.0% for the low performance group. A plot of the DFA results for the three clusters are shown in online Appendix Figure S1. The results of the leave‐one‐out cross validation confirmed the stability of the clusters, with an overall correct classification rate of 87.0%, with correct classification for the high, mid, and low performance groups of 91.4%, 80.7%, 94.0%, respectively. Overall, these results support good consistency for membership to the high and low performance groups and adequate consistency for membership in the mid performance group. A plot of the average cognitive factor scores per each of the three hierarchical clusters and controls is found in Figure 1.

Results of the χ ^2^ analysis evaluating differences in the proportion of participant attrition among the three groups and controls between the baseline and five‐year neuropsychological evaluations was not significant (*p* > 0.05). Overall percent attrition was 63% for the high performance group, 67% for the mid performance group, 75% for the low performance group, and 63% for controls.

### 3.3 Hierarchical cluster cognitive and clinical characteristics

Among the three BP cluster groups, there were significantly different mean scores across the six cognitive factors at baseline (*p* < 0.008), except the high and mid groups did not have significantly different performance on the inhibitory control factor (*p* > 0.008). The high group, in comparison with controls, had significantly better performance on the two memory factors and lower performance on the processing speed with influence resolution factor (*p* < 0.008). The control group performed better than the mid and low groups across the six factors (*p* < 0.008), except for similar performance between controls and the mid group on the inhibitory control factor (*p* > 0.008). These results demonstrate largely unique cognitive performance among the three BP cluster groups. These results are detailed in online Appendix Table S1.

Baseline characteristics of the three BP groups shows that there are significant differences in education, estimated IQ, depression severity, childhood trauma, and number of psychiatric hospitalizations (*p* < 0.05); additionally, there are differences in the expected proportion of group members with a history of a substance use diagnosis, BP‐I diagnosis, and a history of psychosis. Whereas the high group achieved the most education, had the highest estimated IQ, lowest depression severity, lowest reported childhood trauma severity, lowest number of psychiatric hospitalizations, lowest proportion with a history of a substance use disorder, lowest proportion of people diagnosed with BP‐I, and lowest proportion with a history of psychosis; the opposite pattern was generally found for the low group. There were no significant differences among the groups in age of BP onset, mania severity, number of comorbid mental health diagnoses, BMI, number of medical comorbidities, and medication load (p > 0.05). There were also no significant differences between the groups in the proportion of females, history of a suicide attempt, BP‐NOS diagnosis, or use of an antipsychotic medication (p > 0.05). The control group was younger than the three BP groups. The control group also achieved more education and had higher estimated IQ than the mid and low groups. These results are found in Table 1. Additionally, difference in subgroup mood scores, changes in mood scores between neuropsychological assessments, and months since prior neuropsychological evaluation are found in the online Appendices (Data S1).

### 3.4 Unconditional growth model results to determine overall rate of cognitive change

The unconditional growth models identified visual memory as improving over a five‐year period (*p* < 0.05). All other cognitive factors did not have significant rates of change over a five‐year period (*p* > 0.05). The unconditional growth models also identified baseline scores significantly below zero (*p* < 0.01) for all six factors. These results are found in online Appendix Table S2.

### 3.5 Multilevel model results evaluating group differences in rates of cognitive change

**FIGURE 2:**
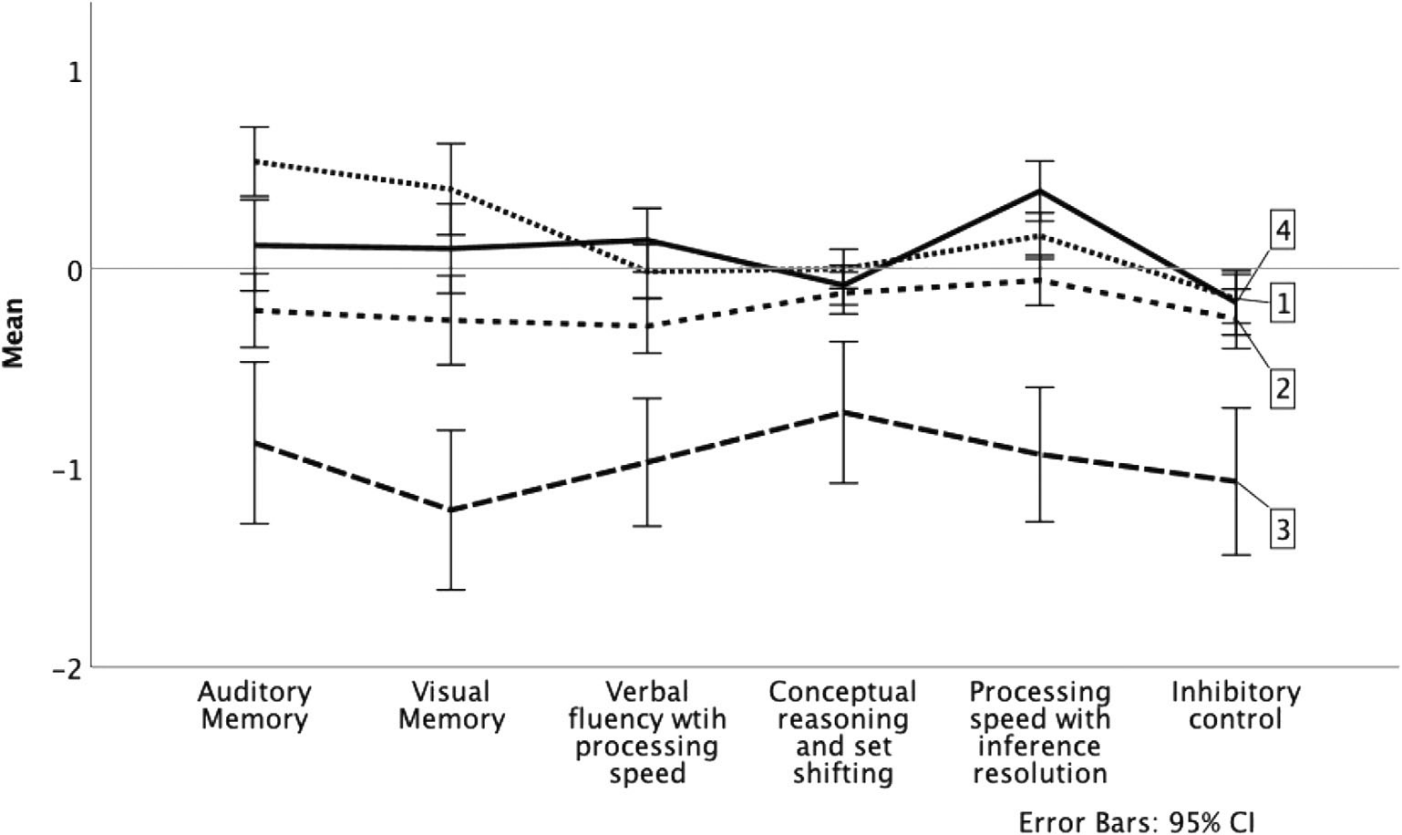
Five‐year cognitive factor means for the three clusters and controls. 1 = high performance group, 2 = mid performance group, 3 = low performance group, 4 = control group

**FIGURE 3:**
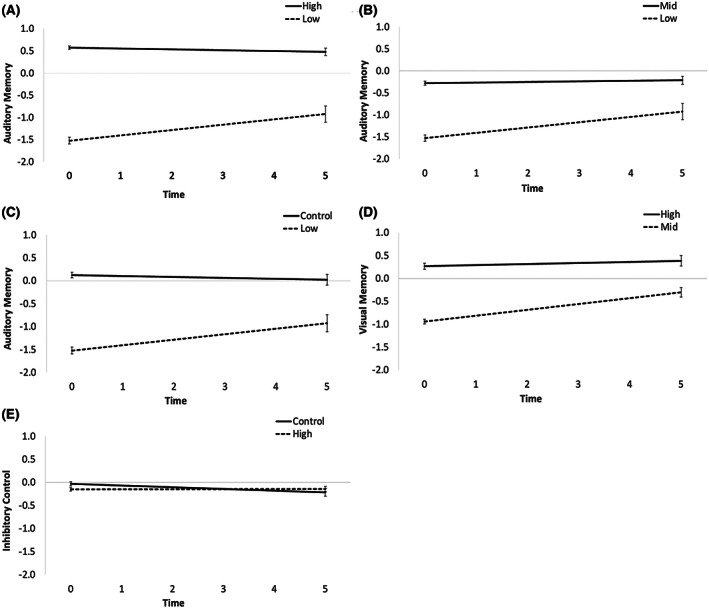
Plot of significant slope results for the between group contrasts. The slopes represent a linear combination of the 0, 1, and 5‐year cognitive factor data. *Y*‐axis is the mean factor score and *x*‐axis is time in years. Error bars are 95% confidence intervals

Results for multilevel analyses that identified significant group differences in slopes (i.e., rate of change) are found in Table 2. Full multilevel analyses results are found in online Appendix Table S3. The low group, in comparison with the high and mid groups, in addition to controls, improved in the auditory memory factor over a five‐year period (*p* < 0.05; see Figure 3A–C for graphical representations of these results). The mid group, in comparison with the high group, improved in the visual memory factor (*p* < 0.05; Figure 3D). These gains in visual memory performance over the five‐year period results in this domain score being consistent with other domain scores at approximately a third of a standard deviation below the mean (see Figure 2 for a visualization of each BP group and controls cognitive performance at the five‐year neuropsychological evaluation). Finally, the high group demonstrated no change in the inhibitory control factor, while controls slightly declined (*p* < 0.05; Figure 3E). Cognitive performance among the three BP groups and controls at the five‐year neuropsychological evaluation demonstrated that the low group's performance remained around one standard deviation below the mean, which was significantly lower than all other groups; while the mid and high groups performed within one half of a standard deviation of the mean, which included minimal differences among the mid and high groups and controls (see online Appendix Table S4).

**TABLE 2:** Significant multilevel model results

Factor		Estimate	Std. error	Sig.
*High versus low performance group*				
Auditory memory	Intercept	0.542	0.041	<0.001
	High versus low	−1.98	0.074	<0.001
	Time	−0.029	0.017	0.104
	High versus low * Time	0.114	0.034	0.001
*High versus mid performance group*				
Visual memory	Intercept	0.228	0.051	<0.001
	High versus mid	−1.089	0.069	<0.001
	Time	0.027	0.019	0.176
	High versus mid * Time	0.095	0.027	<0.001
*Mid versus low performance group*				
Auditory memory	Intercept	−0.252	0.042	<0.001
	Mid versus low	−1.200	0.081	<0.001
	Time	0.008	0.023	0.720
	Mid versus low * Time	0.107	0.048	0.025
*Control versus low performance group*				
Auditory memory	Intercept	0.136	0.054	0.012
	Control versus low	−1.578	0.099	<0.001
	Time	−0.010	0.017	0.557
	Control versus low * Time	0.087	0.035	0.014
*Control versus high performance group*				
Inhibitory control	Intercept	−0.031	0.037	0.403
	Control versus high	−0.133	0.054	0.015
	Time	−0.044	0.012	<0.001
	Control versus high * Time	0.040	0.018	0.024

*Note*: Contrasts are among hierarchical cluster groups and with controls. Intercept = baseline scores, Time = zero, 1‐ and 5‐year data points.

## 4 DISCUSSION

In this study, we identified cognitive subgroups in a large sample of individuals with BP and evaluated these subgroups for differences in longitudinal cognitive trajectory. Consistent with our hypothesis and prior literature,^5^, ^6^, ^7^, ^27^ our sample of those diagnosed with BP fit into three clusters, which we describe as high, mid, and low performance groups. Among the BP subgroups, the high performance group demonstrated a number of clinical characteristics generally found to be associated with better cognitive functioning as compared with the mid and low performance groups.^28^, ^29^, ^30^ Comparisons between the BP subgroups and controls over a five‐year longitudinal course demonstrated that the low performance group improved in auditory memory, while still remaining substantively below all other groups. The mid performance group, as compared with the high performance group, showed improvement in visual memory over the five‐year period. Consistent with our hypothesis, there were minimal differences between the high performance group and controls, with the high performance group demonstrating a slight relative advantage longitudinally in inhibitory control. At the group level, these results are inconsistent with neuroprogression, and demonstrate that cognitively derived BP subgroups and controls have similar longitudinal cognitive changes over a five‐year period.

Consistent with previous studies,^4^, ^6^, ^7^, ^31^ only a minority of participants demonstrated lower cognitive performance. These lower performing participants had a range of demographic, clinical, and mood characteristics, which have been found to be general risk factors and psychiatric risk factors associated with cognitive functioning in BP.^32^, ^33^, ^34^ Two of these characteristics are higher childhood trauma and more psychiatric hospitalizations. Stressors such as these are thought to contribute to an increased allostatic load, with cumulative effects contributing to lower cognitive functioning.^35^ Our baseline findings related to the characteristics of the lower performance group support the hypothesis of a higher allostatic load contributing to lower cognitive functioning. However, these baseline characteristics do not appear to contribute to further cognitive decline.

Over a 5 year period there was no evidence of decline in cognitive functioning for the low performance group, nor for the high or mid performance groups. This lack of neuroprogression in BP is consistent with a recent meta‐analysis, that averaged greater than 5 years of follow‐up, showing those diagnosed with BP generally have similar rates of cognitive changes as the general population.^13^ Furthermore, an additional meta‐analysis demonstrated that those with a recent onset or late life BP demonstrated stable cognitive functioning as compared with controls,^16^ which suggests that cognition is affected during the neurodevelopmental period rather than following a neuroprogressive course. These meta‐analyses included longitudinal studies of five or more years with BP participants diagnosed with BPI,^34^ mixed BPI and BPII,^10^, ^36^ and participants in later life.^14^ Our cognitive subgroups similarly did not evince cognitive decline, providing further support that BP is not inherently a neuroprogressive condition.

It should be noted that consistent with the allostatic load hypothesis,^35^ there is some indication that those who experience more frequent manic and/or hypomanic episodes may be more likely to experience a neuroprogressive course,^10^ though not all studies support this theory.^36^ These mixed findings highlight the importance of studies, such as this, that parse BP participants into meaningful subgroups that extend beyond traditional diagnostic categories. There is a subset of individuals diagnosed with BP who experience greater lifetime allostatic load and may present with a neuroprogressive course. Following BP participants who are experiencing a greater allostatic load remains a vital undertaking to understand and help those with a likely more severe course. In addition, as attrition is not inherently random, it is also possible that those with greater neuroprogression were lost to follow‐up.

This study has a few limitations. First, our participants with BP and controls tended to be middle aged, have higher educational attainment, higher estimated intelligence, were capable of staying in this longitudinal study over 5 years, and were skewed towards females. In particular, higher intellectual functioning may be a protective factor against cognitive decline in BP.^37^ As such, this may somewhat limit the generalizability of our findings to the larger population of individuals with BP, our longitudinal findings were generally consistent with prior studies showing minimal longitudinal changes as compared with controls.^13^, ^16^ Second, we used cognitive domains originally derived from factor analysis in our analyses to reduce the number of data points rather than individual test variables. While a certain level of specificity can be lost, the mean age‐normed z‐scores for each cognitive factor can provide robust and easily understood cognitive domains. Lastly, the relative longitudinal improvements in auditory and visual memory scores may simply represent regression to the mean with minimal overall significance.

With the continued growth of longitudinal studies of BP, such as our own Longitudinal Study of Bipolar Disorder or the Global Bipolar Cohort Study, and the International Consortium Investigating Neurocognition in Bipolar Disorder,^27^ changes in cognition can be studied over an increasingly longer period of time and in larger sample sizes. Increasing the time period of follow‐up will be important to appreciate the natural course and impact of cognition impairments in people diagnosed with BP. A five‐year follow‐up may not be a sufficient duration to observe decrements in these cognitive domains. In part, the sample characteristics demonstrated that the cognitive subgroups have different clinical features, such as levels of childhood trauma, depression, and number of psychiatric hospitalizations. As this is a naturalistic, incidental enrollment, lifespan study, we did not address the role of these features in the work. Prior studies by our group with this sample do show relations of child trauma, work history, number hospitalizations, episodes per year ill, and to a lesser extent, symptoms with cognition.^19^, ^38^, ^39^ Replication of these findings in other samples is particularly important given our sample characteristics. Further, longitudinal studies would support Kapczinski et al.'s^12^ staging model if changes in cognition coincide with changes in functional status, particularly for the low performance group who would be predicted to have lower functional status.

In summary, our sample of individuals diagnosed with BP support three cognitive subgroups, with average cognitive functioning demonstrated by most participants. The high cognitive functioning group had a number of characteristics related to better cognitive functioning as compared with the mid and lower performance groups, that included a range of demographic, clinical, and mood characteristics. The cognitive change patterns of the mid and low performance groups did not impact their outcomes. Our findings provide further support to the heterogeneity of cognitive functioning in BP and support the value in parsing BP by cognitive functioning to provide greater understanding of the unique characteristics and cognitive trajectories of people with BP.

## CONFLICT OF INTEREST

Dr. McInnis has consulted for Janssen and Otsuka Pharmaceuticals in the past 3 years. Ehrlich, Ryan, Burdick, Langenecker, and Marshall, have no conflicts to disclose.

### PEER REVIEW

The peer review history for this article is available at https://publons.com/publon/10.1111/acps.13460.

## Supporting information


**Appendix S1**. Supporting Information.

## Data Availability

The data that support the findings of this study are available from the corresponding author upon reasonable request.
