# Supplementary material for: Cognitive subgroups and their longitudinal trajectories in bipolar disorder
Source: Acta Psychiatr Scand. 2022 Jun 25;146(3):240–50. doi: 10.1111/acps.13460 (PMC9545624; doi:10.1111/acps.13460)
Supplement: Supplementary file 1 — Appendix S1. Supporting Information. [file ACPS-146-240-s001.docx]

**Appendices**

**Diagnostic update**

Updated diagnostic data was available at four years post study entry, which was similarly determined by a two-person review and consensus. At four years post study entry, a total of nine of the 568 BP participants had a diagnostic change; five BPII participants were updated to BPI, two BP-NOS participants were updated to BPI, one BPI participant was updated to BPII, and one BP-NOS participant was updated to Major Depressive Disorder.

**Cognitive factor**

The auditory memory factor includes six subtest scores (total learning five trials, short delay free recall, short delay cued recall, long delay free recall, long delay cued recall, and recognition hits) of the California Verbal Learning Test, Second Edition (CVLT-II)^1^. The visual memory factor includes three subtest scores (immediate recall, delayed recall, and recognition) of the Rey-Osterrieth Complex Figure Test^2^. The verbal fluency with processing speed factor includes the following six subtest scores: FAS verbal fluency task of the Controlled Oral Word Association Test and Animal Fluency^3^, Digit Symbol-Coding from the Wechsler Adult Intelligence Scale-Third Edition^4^, Stroop Color and Word Test^5^, and Trail Making Test – Form B^6^. The conceptual reasoning and set-shifting factor include three subtest scores, two from the Wisconsin Card Sorting Test^7^, number of correct cards sorted and perseverative errors (inverted), and Parametric Go/No-Go^8^ mean accuracy of target trials. The processing speed with inference resolution factor includes five subtest scores, Trail Making Test – Forms A and B, Digit Symbol Coding Test, Stroop interference trial, and Parametric Go/No-Go mean target response time (inverted). Finally, the inhibitory control factor includes Parametric Go/No-Go mean target response time (inverted) and Parametric Go/No-Go mean accuracy for the inhibitory trials.

Tests with alternative versions included the CVLT-II^1^, Rey-Osterrieth Complex Figure Test^2^, FAS verbal fluency task of the Controlled Oral Word Association Test and Animal Fluency^3^, Digit Symbol-Coding from the Wechsler Adult Intelligence Scale-Third Edition^4^, and the Trail Making Test- Forms A & B^6^.

**Differences in subgroup mood scores and time between neuropsychological assessment**

At the one-year neuropsychological assessment, the low group continued to demonstrate higher depression severity than the mid and high groups (p < .005). However, at the five-year neuropsychological assessment, there were no significant differences in depression severity among the BP groups (p > .05). At both one- and five-year neuropsychological assessments, there were no significant differences in mania severity among the BP groups (p > .05). Among the baseline, one- and five-year neuropsychological assessments, the high and mid groups did not demonstrate a significant change in depression or mania severity. However, the low group declined in depression severity between the baseline and five-year neuropsychological assessment (p < .01). Of note, this decline in depression severity for the low group totaled 3.54 points on the HAMD. Additionally, there were no statistical differences among the four groups in the number of months that elapsed between the baseline, one- and five-year neuropsychological evaluations (p > .05).

**Table 1.** *Mean baseline cognitive factor scores for hierarchical cluster groups and controls.*

|  | (1) High | (2) Mid | (3) Low | (4) Controls |
| --- | --- | --- | --- | --- |
| Auditory memory | .574 (.531) | - .279 (.744) | - 1.523 (.739) | - .093 (.971) |
| Visual memory | .266 (.725) | - .941 (.786) | - 1.629 (.568) | - .313 (.998) |
| Verbal fluency with processing speed | .019 (.545) | - .327 (.587) | - 1.078 (.563) | .102 (.626) |
| Conceptual reasoning and set shifting | .027 (.524) | - .235 (.622) | - .845 (.898) | - .043 (.512) |
| Processing speed with influence resolution | .112 (.492) | - .247 (.628) | - .904 (.618) | .265 (.612) |
| Inhibitory control | - .148 (.531) | - .299 (.729) | - .775 (.655) | - .240 (.675) |

*Note.* Data is provided as mean (standard deviation) for each of the six cognitive factors.

**Table 2.** *Unconditional means model results.*

| Factor |  | Estimate | Std. Error | Sig. |
| --- | --- | --- | --- | --- |
| Auditory memory | Intercept | - .075 | .027 | .006 |
|  | Time | .011 | .016 | .489 |
| Visual memory | Intercept | - .475 | .029 | < .001 |
|  | Time | .082 | .017 | < .001 |
| Verbal fluency with processing speed | Intercept | - .200 | .019 | < .001 |
|  | Time | .015 | .021 | .470 |
| Conceptual reasoning and set shifting | Intercept | - .133 | .018 | < .001 |
|  | Time | .005 | .009 | .607 |
| Processing speed with inference resolution | Intercept | - .073 | .019 | < .001 |
|  | Time | .025 | .021 | .232 |
| Inhibitory control | Intercept | - .236 | .019 | < .001 |
|  | Time | - .014 | .011 | .220 |

*Note.* Results for the total sample of 823 participants. Intercept = average baseline performance, Time = rate of change (i.e., slope).

**Table 3.** *Multilevel analyses results.*

| Factor |  | Estimate | Std. Error | Sig. |
| --- | --- | --- | --- | --- |
| High versus low performance group | |  |  |  |
| Auditory memory | Intercept | .542 | .041 | < .001 |
|  | High vs low | - 1.98 | .074 | < .001 |
|  | Time | - .029 | .017 | .104 |
|  | High vs low * Time | .114 | .034 | .001 |
| Visual memory | Intercept | .228 | .046 | < .001 |
|  | High vs low | -1.777 | .081 | < .001 |
|  | Time | .024 | .021 | .258 |
|  | High vs low * Time | .078 | .041 | .058 |
| Conceptual reasoning with set shifting | Intercept | .063 | .036 | .080 |
|  | High vs low | - .772 | .064 | < .001 |
|  | Time | - .009 | .017 | .572 |
|  | High vs low * Time | .025 | .033 | .453 |
| Processing speed with inference resolution | Intercept | .099 | .036 | .006 |
|  | High vs low | - .955 | .064 | < .001 |
|  | Time | .015 | .011 | .152 |
|  | High vs low * Time | - .015 | .022 | .490 |
| Verbal fluency with processing speed | Intercept | .018 | .037 | .621 |
|  | High vs low | - 1.069 | .066 | < .001 |
|  | Time | - .009 | .010 | .355 |
|  | High vs low * Time | .029 | .020 | .148 |
| Inhibitory control | Intercept | - .166 | .039 | < .001 |
|  | High vs low | - .564 | .069 | < .001 |
|  | Time | - .004 | .013 | .788 |
|  | High vs low * Time | - .047 | .026 | .074 |
| High versus mid performance group | |  |  |  |
| Auditory memory | Intercept | .536 | .041 | < .001 |
|  | High vs mid | - .787 | .055 | < .001 |
|  | Time | -.021 | .023 | .359 |
|  | High vs mid * Time | .028 | .032 | .376 |
| Visual memory | Intercept | .228 | .051 | < .001 |
|  | High vs mid | - 1.089 | .069 | < .001 |
|  | Time | .027 | .019 | .176 |
|  | High vs mid * Time | .095 | .027 | < .001 |
| Conceptual reasoning with set shifting | Intercept | .063 | .031 | .044 |
|  | High vs mid | - .251 | .042 | < .001 |
|  | Time | - .009 | .014 | .509 |
|  | High vs mid * Time | .029 | .019 | .138 |
| Processing speed with inference resolution | Intercept | .099 | .038 | .010 |
|  | High vs mid | - .321 | .052 | < .001 |
|  | Time | .016 | .009 | .100 |
|  | High vs mid * Time | .004 | .013 | .772 |
| Verbal fluency with processing speed | Intercept | .019 | .038 | .623 |
|  | High vs mid | - .342 | .051 | < .001 |
|  | Time | - .009 | .009 | .317 |
|  | High vs mid * Time | .008 | .013 | .529 |
| Inhibitory control | Intercept | - .165 | .042 | < .001 |
|  | High vs mid | - .129 | .056 | .022 |
|  | Time | - .004 | .03 | .776 |
|  | High vs mid * Time | - .008 | .017 | .642 |
| Mid versus low performance group | |  |  |  |
| Auditory memory | Intercept | - .252 | .042 | < .001 |
|  | Mid vs low | - 1.200 | .081 | < .001 |
|  | Time | .008 | .023 | .720 |
|  | Mid vs low * Time | .107 | .048 | .025 |
| Visual memory | Intercept | - .859 | .045 | < .001 |
|  | Mid vs low | - .686 | .087 | < .001 |
|  | Time | .121 | .018 | < .001 |
|  | Mid vs low * Time | - .028 | .038 | .472 |
| Conceptual reasoning with set shifting | Intercept | - .188 | .035 | < .001 |
|  | Mid vs low | - .519 | .068 | < .001 |
|  | Time | .019 | .017 | .253 |
|  | Mid vs low * Time | - .006 | .035 | .851 |
| Processing speed with inference resolution | Intercept | -.222 | .038 | < .001 |
|  | Mid vs low | - .634 | .072 | < .001 |
|  | Time | .020 | .011 | .065 |
|  | Mid vs low * Time | - .021 | .023 | .368 |
| Verbal fluency with processing speed | Intercept | -.323 | .035 | < .001 |
|  | Mid vs low | - .726 | .066 | < .001 |
|  | Time | - .002 | .009 | .867 |
|  | Mid vs low * Time | .019 | .019 | .320 |
| Inhibitory control | Intercept | - .294 | .041 | < .001 |
|  | Mid vs low | - .436 | .078 | < .001 |
|  | Time | - .011 | .014 | .441 |
|  | Mid vs low * Time | - .039 | .029 | .188 |
| Control versus low performance group | |  |  |  |
| Auditory memory | Intercept | .136 | .054 | .012 |
|  | Control vs low | - 1.578 | .099 | < .001 |
|  | Time | -.010 | .017 | .557 |
|  | Control vs low * Time | .087 | .035 | .014 |
| Visual memory | Intercept | - .265 | .057 | < .001 |
|  | Control vs low | - 1.283 | .105 | < .001 |
|  | Time | .051 | .017 | .004 |
|  | Control vs low * Time | .042 | .035 | .232 |
| Conceptual reasoning with set shifting | Intercept | - .010 | .040 | .803 |
|  | Control vs low | - .722 | .074 | < .001 |
|  | Time | - .015 | .012 | .204 |
|  | Control vs low * Time | .036 | .024 | .133 |
| Processing speed with inference resolution | Intercept | .275 | .039 | < .001 |
|  | Control vs low | - 1.132 | .071 | < .001 |
|  | Time | .017 | .009 | .083 |
|  | Control vs low * Time | - .017 | .020 | .407 |
| Verbal fluency with processing speed | Intercept | .124 | .039 | .002 |
|  | Control vs low | - 1.176 | .071 | < .001 |
|  | Time | .001 | .008 | .939 |
|  | Control vs low * Time | .016 | .018 | .372 |
| Inhibitory control | Intercept | - .031 | .041 | .451 |
|  | Control vs low | - .700 | .075 | < .001 |
|  | Time | - .044 | .015 | .003 |
|  | Control vs low * Time | - .004 | .030 | .884 |
| Control versus mid performance group | |  |  |  |
| Auditory memory | Intercept | .136 | .045 | .002 |
|  | Control vs mid | - .386 | .061 | < .001 |
|  | Time | - .017 | .028 | .544 |
|  | Control vs mid * Time | .021 | .039 | .584 |
| Visual memory | Intercept | - .247 | .048 | < .001 |
|  | Control vs mid | - .602 | .067 | < .001 |
|  | Time | .055 | .029 | .066 |
|  | Control vs mid * Time | .075 | .041 | .072 |
| Conceptual reasoning with set shifting | Intercept | - .009 | .030 | .751 |
|  | Control vs mid | - .179 | .041 | < .001 |
|  | Time | - .016 | .014 | .259 |
|  | Control vs mid * Time | .035 | .019 | .070 |
| Processing speed with inference resolution | Intercept | .289 | .032 | < .001 |
|  | Control vs mid | - .514 | .044 | < .001 |
|  | Time | .026 | .019 | .196 |
|  | Control vs mid * Time | .006 | .028 | .829 |
| Verbal fluency with processing speed | Intercept | .125 | .038 | .001 |
|  | Control vs mid | - .448 | .053 | < .001 |
|  | Time | .001 | .009 | .987 |
|  | Control vs mid * Time | - .002 | .012 | .862 |
| Inhibitory control | Intercept | - .031 | .042 | .450 |
|  | Control vs mid | - .262 | .057 | < .001 |
|  | Time | - .044 | .013 | .001 |
|  | Control vs mid * Time | .033 | .019 | .086 |
| Control versus high performance group | |  |  |  |
| Auditory memory | Intercept | .135 | .047 | .004 |
|  | Control vs high | .412 | .068 | < .001 |
|  | Time | - .009 | .015 | .560 |
|  | Control vs high * Time | - .024 | .022 | .260 |
| Visual memory | Intercept | - .265 | .056 | < .001 |
|  | Control vs high | .495 | .081 | < .001 |
|  | Time | .051 | .018 | .005 |
|  | Control vs high * Time | - .024 | .026 | .350 |
| Conceptual reasoning with set shifting | Intercept | - .009 | .026 | .715 |
|  | Control vs high | .072 | .038 | .059 |
|  | Time | - .015 | .013 | .224 |
|  | Control vs high * Time | .006 | .018 | .750 |
| Processing speed with inference resolution | Intercept | .289 | .029 | < .001 |
|  | Control vs high | - .194 | .042 | < .001 |
|  | Time | .027 | .019 | .156 |
|  | Control vs high * Time | - .018 | .027 | .507 |
| Verbal fluency with processing speed | Intercept | .124 | .038 | .001 |
|  | Control vs high | - .104 | .055 | .057 |
|  | Time | .001 | .008 | .912 |
|  | Control vs high * Time | - .009 | .012 | .411 |
| Inhibitory control | Intercept | - .031 | .037 | .403 |
|  | Control vs high | - .133 | .054 | .015 |
|  | Time | - .044 | .012 | < .001 |
|  | Control vs high * Time | .040 | .018 | .024 |

*Note.* Contrasts between hierarchical cluster groups and with controls. Intercept = baseline scores, Time = zero, 1- and 5-year data points.

**Table 4.** *Mean five-year cognitive factor scores for the hierarchical cluster groups and controls.*

|  | (1) High | (2) Mid | (3) Low | (4) Controls |
| --- | --- | --- | --- | --- |
| Auditory memory | .483 (.789) | - .211 (.865) | - .922 (.988) | .024 (1.112) |
| Visual memory | .386 (1.029) | - .301 (1.024) | - 1.169 (.977) | - .024 (1.097) |
| Verbal fluency with processing speed | - .035 (.592) | - .303 (.642) | - .937 (.771) | .108 (.732) |
| Conceptual reasoning and set shifting | - .007 (.437) | - .125 (.481) | - .772 (.862) | - .110 (.472) |
| Processing speed with influence resolution | .166 (.512) | - .053 (.581) | - .934 (.816) | .387 (.682) |
| Inhibitory control | - .142 (.538) | - .292 (.721) | - 1.061 (.876) | - .214 (.758) |

*Note.* Data is provided as mean (standard deviation) for each of the six cognitive factors. Significant differences were computed with ANOVA analyses testing for differences between the groups average baseline performance with a Bonferroni corrected for six comparisons p = .008. For the auditory memory factor, there were significant group differences across all group comparisons except for the comparison between group 2 and 4. For the visual memory factor, there were significant group differences across all comparisons except for the comparisons between group 1 and 4, and between 2 and 4. For the verbal fluency with processing speed factor, there were significant group differences across all comparisons except for the comparison between group 1 and 4. For the conceptual reasoning and set shifting factor, there were significant group differences for the comparisons between groups 1 and 3, 2 and 3, in addition to 3 and 4. For the processing speed with inference resolution factor, there were significant group differences across all comparisons except for the comparisons between group 1 and 2, and between 1 and 4. The inhibitory control factor had significant group differences for the comparisons between groups 1 and 4, 1 and 2, in addition to 2 and 4.

**Figure 1.** *Canonical discriminant functions*

Results of the discriminant function analysis for the three hierarchical cluster groups along with the group centroid. 1 = high performance group, 2 = mid performance group, 3 = low performance group.

References

1. Delis DC, Kramer JH, Kaplan E, Ober BA. California verbal learning test. *Assessment*. 2000;

2. Meyers JE, Meyers KR. *Rey Complex Figure Test and recognition trial professional manual*. Psychological Assessment Resources; 1995.

3. Benton A, Hamsher K. Multilingual aphasia examination manual. *Iowa City: University of Iowa*. 1978;

4. Wechsler D. *WAIS-III*. Psychological Corporation San Antonio, TX; 1997.

5. Golden CJ, Freshwater SM. Stroop color and word test. 1978;

6. Armitage SG. An analysis of certain psychological tests used for the evaluation of brain injury. *Psychological monographs*. 1946;60(1):i.

7. Grant DA, Berg E. A behavioral analysis of degree of reinforcement and ease of shifting to new responses in a Weigl-type card-sorting problem. *Journal of experimental psychology*. 1948;38(4):404.

8. Langenecker SA, Bieliauskas LA, Rapport LJ, Zubieta J-K, Wilde EA, Berent S. Face emotion perception and executive functioning deficits in depression. *Journal of clinical and experimental neuropsychology*. 2005;27(3):320-333.
